# Using Digital Platform Approach to Reduce Salt Intake in a Sample of UAE Population: An Intervention Study

**DOI:** 10.3389/fpubh.2022.860835

**Published:** 2022-05-24

**Authors:** Amjad H. Jarrar, Ayesha S. Al Dhaheri, Helen Lightowler, Leila Cheikh Ismail, Fatima Al-Meqbaali, Mo'ath F. Bataineh, Aseilah Alhefeiti, Maithah Albreiki, Nouf Albadi, Salama Alkaabi, Pariyarath S. Thondre

**Affiliations:** ^1^Department of Nutrition and Health, College of Medicine and Health Sciences, United Arab Emirates University (UAEU), Al Ain, United Arab Emirates; ^2^Oxford Brookes Center for Nutrition and Health, Faculty of Health and Life Sciences, Oxford Brookes University, Oxford, United Kingdom; ^3^Department of Clinical Nutrition and Dietetics, College of Health Sciences, University of Sharjah, Sharjah, United Arab Emirates; ^4^Nuffield Department of Women's & Reproductive Health, University of Oxford, Oxford, United Kingdom; ^5^Department of Sport Rehabilitation, Faculty of Physical Education and Sport Science, Hashemite University, Zarqa, Jordan

**Keywords:** social media, intervention, urinary sodium and potassium excretion, Knowledge, attitude and practice (KAP), digital platform

## Abstract

**Background:**

Non-communicable diseases (NCDs) are the leading causes of mortality globally, accounting for more deaths than all other causes combined. World Health Organization launched its initiative in 2013 to reduce the intake of salt, the number of countries that have national sodium reduction strategies reached to 89 countries in 2017. In 2020, a study conducted in UAE showed more than 65% of the population exceeded WHO recommendations for salt intake. This study aimed to measure effectiveness of using digital platform approach to deliver educational materials to facilitate salt reduction in a sample of UAE population.

**Methods:**

A controlled parallel intervention study was conducted in 2020. A sample of 121 participants completed the study and fulfilled the inclusion criteria with female to male ratio of (0.95:1.05). Participants were distributed randomly into three groups Control group, WhatsApp group, and Electronic Brochures group. Educational materials were distributed among participants of WhatsApp and Electronic brochures groups for 6-weeks. 24-h urinary excretion for sodium, potassium and creatinine, were measured in addition to KAP questionnaire and physical activity on two occasions at baseline and endpoint after 10-weeks (6-weeks of educational intervention).

**Results:**

Both intervention groups showed a reduction in sodium with 278 mg (*p* < 0.001) for WhatsApp group (*n* = 41) and 169 mg (*p* < 0.018) for Electronic brochures group (*n* = 41), while Control group didn't show any significant change. Moreover, the percentage of participants exceeding WHO recommendation of sodium intake was significantly reduced at the end of intervention, (*p* = 0.004). WhatsApp group was more efficient in the percentage of reduction of participants exceeding WHO recommendation compared with baseline, with *p* = 0.023. A significant reduction in the practice toward adding salt during cooking, use of table salt, adding salt before tasting the foods and use of chicken stocks for both intervention groups was noted with *p* < 0.05. Intervention groups showed a significant improvement (*p* < 0.001) in Food and Health related knowledge after 6-weeks of intervention.

**Conclusion:**

The digital platform approach such as WhatsApp and Electronic Brochure were effective in salt reduction. This study proves that UAE population is ready to reduce salt intake with appropriate education materials and easy delivery approach.

## Introduction

Noncommunicable diseases (NCDs) are the leading causes of mortality globally, accounting for more deaths than all other causes combined. Nearly 80% of NCD-related deaths occur in the low- and middle-income countries (LMICs) (1). In the Eastern Mediterranean Region, it is estimated that NCDs account for over 50% of annual deaths (2.2 million deaths) and 60% of the disease burden (2, 3).

It is projected that deaths from NCDs will increase by 25% in the Region, recording the second highest projected increase among the 6 WHO regions (2). In fact, it is estimated that NCDs cause between 65 and 78% of deaths in the Gulf Cooperation Council (GCC) (Bahrain, Kuwait, Oman, Qatar, Saudi Arabia, and the United Arab Emirates (UAE) (4, 5), specifically, the prevalence of cardiovascular diseases (CVDs), which is rapidly growing, constitute the main underlying causes of morbidity and mortality in countries of the Eastern Mediterranean Region (2, 6).

High blood pressure is recognized as a major underlying risk factor for CVDs (7). According to WHO, it is estimated that 62% of all strokes and 49% of coronary heart disease events are secondary to high blood pressure (8, 9). Globally in 2017, high sodium intake led to ~3 million deaths and loss of 70 million disability-adjusted life-years (7).

A key factor in the success of efforts to reduce sodium intake is that salt (sodium chloride), the primary source of sodium in the diet—has desirable characteristics from a culinary perspective.

Many Asian countries that have documented sodium sources consume most of their sodium from discretionary food sources, accounting for most dietary sodium intake. In the People's Republic of China, most (76%) dietary sodium was from salt added in home cooking, about 50% less in southern than northern areas of the country. In Japan, most (63%) dietary sodium came from soy sauce (20%), commercially processed fish/seafood (15%), salted soups (15%), and preserved vegetables (13%) (10).

In the United States, the United Kingdom and Australia most (95%) of dietary sodium came from processed foods, including breads/cereals/grains (6).

WHO has recommended different strategies approach to reduce salt intake at country levels, these includes; consumer education and awareness food reformulation, Front-of-pack labeling, and salt taxation (2, 7, 11). The number of countries that have national sodium reduction strategies doubled from 2010 to 2014 (7, 11). As of 2016–2017, 89 countries have national policies that support sodium reduction (5, 7). Although the specific strategies vary, almost all take a multi-component approach. Consumer education is the most common intervention, although an increasing number incorporate a regulatory approach as well (7, 11).

Strategies should be appropriate to the local context in terms of the sources of dietary sodium, existing regulatory mechanisms, and access to resources. No single strategy is enough to reach the WHO goal of a 30% reduction in sodium intake by 2025 (7).

Two cross sectional studies conducted in UAE one among adult population (4) and other one among University student (12). Both studies showed KAP toward salt intake was poor and most of the participants failed to recognized foods with high salt contents. Consumer awareness strategy recommended by WHO for salt reduction (2), promote Social Cognitive Learning Theory, which acknowledges the constant interaction that exists between the individual and his or her environment, both structural and social, to shape behavior (11, 13).

To date, there is no published intervention study conducted in the UAE that has used urinary sodium excretion and knowledge, attitude, and practice (KAP) to assess salt intake before and after an educational intervention. Therefore, this parallel study aimed to assess the efficiency and effectiveness of two different intervention approaches for 6-weeks (WhatsApp and electronic-brochures) to reduce sodium intake.

## Materials and Methods

This study obtained ethical approval from the University Research Ethics Committee at Oxford Brookes University (UREC), Number: 191337. In addition to, ethical approval from United Arab Emirates University (UAEU) Research Ethics Committee (ERS_2020_6107). This study was conducted according to the stated principles in the Declaration of Helsinki (14). All subjects gave written informed consent to participate in the study, and all the communication were online as a response to COVID-19.

### Study Design and Participants

A controlled intervention parallel design study comparing the efficiency and effectiveness of two different educational intervention approaches (WhatsApp and electronic brochures) to reduce sodium intake, with a control group receiving no educational materials for the intervention period. The primary outcomes of the study were urinary sodium, potassium, knowledge attitude and practice (KAP), physical activity. All these outcomes measured in two occasions at baseline and at endpoint after 10-weeks (6 weeks of educational intervention).

The sample size was determined based on the WHO Eastern Mediterranean Region Office (WHO-EMRO) protocol published in 2010 for 24-h urine collection and analysis (13). Participants were divided into two age groups, 20–29.9, and 30–40. To be able to detect a difference in 20 mmol/day of sodium (~459 mg of sodium) with 25 mmol standard deviations, the sample size needed for each age stratum was 32 participants from both genders. Therefore, for the three intervention groups, 96 participants, (alpha = 0.05, power = 0.80), were recommended. To account for attrition—e.g., non-participation, incomplete collection, or implausible values—which may be as high as 20%, up to 115 participants from all age and gender strata should be recruited (13).

The parallel intervention study was conducted from October 2020–January 2021 in the United Arab Emirates. A random sample of 148 healthy individuals aged between 20 and 40 years were recruited to participate in the study from the three geographical areas of UAE (Western, Northern, and Eastern region). Participants were randomly distributed into the three groups (Control, WhatsApp, and Electronic Brochure) using Altman and Bland (15) procedure. RAND() function was generated according to gender-age group and the three intervention groups.

Several different methods were used for recruitment: email circulation to students and staff (Non-medical students and staff) from Applied Sciences and Humanities Colleges members of UAE University, and social media approach such as WhatsApp and Instagram for population out UAEU.

Inclusion criteria at screening were participants aged 20 to 40 years for both gender, non-pregnant, and non-lactating, no known chronic kidney disease, renal failure, hypertension with medications, and liver diseases, no medical condition(s) or medication(s) known to affect urination and ability to collect 24-h urine. While, the exclusion criteria at screening were those with chronic diseases (i.e., heart disease, hypertension on medications, renal failure, liver disease), pregnant and lactating women, those on diuretics, and women who had their menstrual cycle during the time of urine collection and those who had a positive COVID-19 test. Exclusion criteria after urine collection included those that were unable to collect adequate urine within the 24-h period (i.e., volume <500 ml). In addition to that, creatinine level of the reference range 500 to 2,000 mg/day was used in the current study, which is equivalent to 9–26 mg/kg of body mass for female participants and 13–29 mg/kg of body mass for male participants (4, 16, 17).

### Anthropometric Measurements

Body weight and height were measured for each participant and their body mass index (BMI) was calculated as weight (kg) divided by height (m) squared (kg/m^2^). Height was recorded to the nearest 1 cm using a stadiometer (Seca Stadiometer, Seca Ltd., Birmingham, UK) and weight was recorded using a balance (Seca Stadiometer, Seca Ltd., Birmingham, UK) to the nearest 0.1 kg (18). All the precaution requirements implemented by UAEU and by Federal Government of UAE were performed on the participants, which included checking body temperature prior to entering nutrition clinics, physical distancing and space capacity. In addition, a valid negative test for COVID-19 for 30 days.

### Knowledge, Attitude, and Practice (KAP) Questionnaire

Participants were asked to complete the self-reported questionnaire on two occasions, one at baseline and second time after 10-weeks (including 6-weeks of educational intervention). The questionnaire assessed knowledge related to salt and health outcomes, frequency of consumption and their perceived salt consumption. The Validated KAP questionnaire was obtained from WHO/PAHO protocol for the assessment of population sodium intake and behaviors (13). The development and performance of the specific questionnaire has been described elsewhere (4, 12). The KAP questionnaire was used to in two cross sectional studies in UAE population (4) and among University students (12).

### Educational Materials

In the intervention stage, educational materials were shared with the two-intervention groups, aimed to improve the knowledge of the participants to reduce salt intake, highlight the health hazards of high salt intake, importance of checking food labels, and alternatives for salt.

Educational materials were developed using information from Weqaya program—Health Authority of Abu Dhabi—HAAD-UAE, https://weqaya.doh.gov.ae/, Central for Disease and Control Prevention (CDC—Salt https://www.cdc.gov/salt/index.htm. In addition, the information obtained from the outcome of the cross-sectional study conducted in UAE (4). For the WhatsApp group, every 7-days a new message was delivered for 6 weeks, with a new topic every week. Every 3-days the same message was repeated. While for the electronic brochure group, every 2 weeks a new electronic brochure with a new topic was sent to the participants by email.

### 24-h Urine Collections and Analysis

A single timed 24-h urine collection was obtained for the estimation of sodium excretion. Participants were given written and verbal instructions for the 24-h urine collection procedure. A 3-L coded plastic bottle was given to each participant for urine collection. Participants were asked to discard the first urine of the day and to collect all urine in the plastic bottle provided over the following 24-h. Participants were also asked to write on a separate sheet the time and date at the start and end of the urine collection, indicating occasions they missed urination. Urine analysis for sodium, potassium, and creatinine levels were conducted in the College of Agriculture & Veterinary Medicine (CAVM) laboratories at United Arab Emirates University (UAEU). For the measurement of sodium and potassium levels in the urine, 50 mL of the urine sample was mixed with 200 μL of 1% nitric acid. Analytical solutions were introduced to a Varian ICP-OES model 710-ES spectrometer for sodium and potassium measurements (19). 24-h urinary creatinine was measured using ab204537 Creatinine Assay Kit based on colorimetric Assay (20), with UV-Visible spectrophotometer (Multiscan Go, Thermo-Fisher Scientific, MA, USA). The assay relies on Jaffe‘ reaction (20). The measurements were taken on two occasions at baseline and after 10 weeks (including 6-weeks of educational materials).

### Statistical Analysis

Data were recorded and analyzed using the Statistical Package for Social Sciences (SPSS) software, version 25 (SPSS, Chicago, IL, USA). Normality of data across combination of independent variables was tested using Shapiro Wilks. Homogeneity of data was tested using Leven's test. Continuous variables were presented as mean ± standard deviation and categorical variables were expressed as numbers and percentages. Descriptive statistics were used to summarize the baseline characteristics of the study participants.

Mixed repeated measures ANOVA test was performed to assess the main effects of time (baseline vs. endpoint) and intervention groups (Control, WhatsApp, and Electronic Brochures) on 24-h urinary sodium and potassium, and creatinine excretions. For interaction analysis, One-way ANOVA, followed by Bonferroni *post-hoc* test, and dependent *t*-test were used to compare dependent variables according to time and intervention groups. One-sample *t*-test was used to compare the mean urinary sodium, salt, and potassium excretions with the recommended dietary allowance, and assessment was carried out based on gender using independent *t*-test. The effect size was calculated as Cohen *d* for *t*-test and partial eta-squared ($\eta_{p}^{2}$) for ANOVA. *P*-values < 0.05 were considered statistically significant.

## Results

### Sample Characteristics

**Figure 1:**
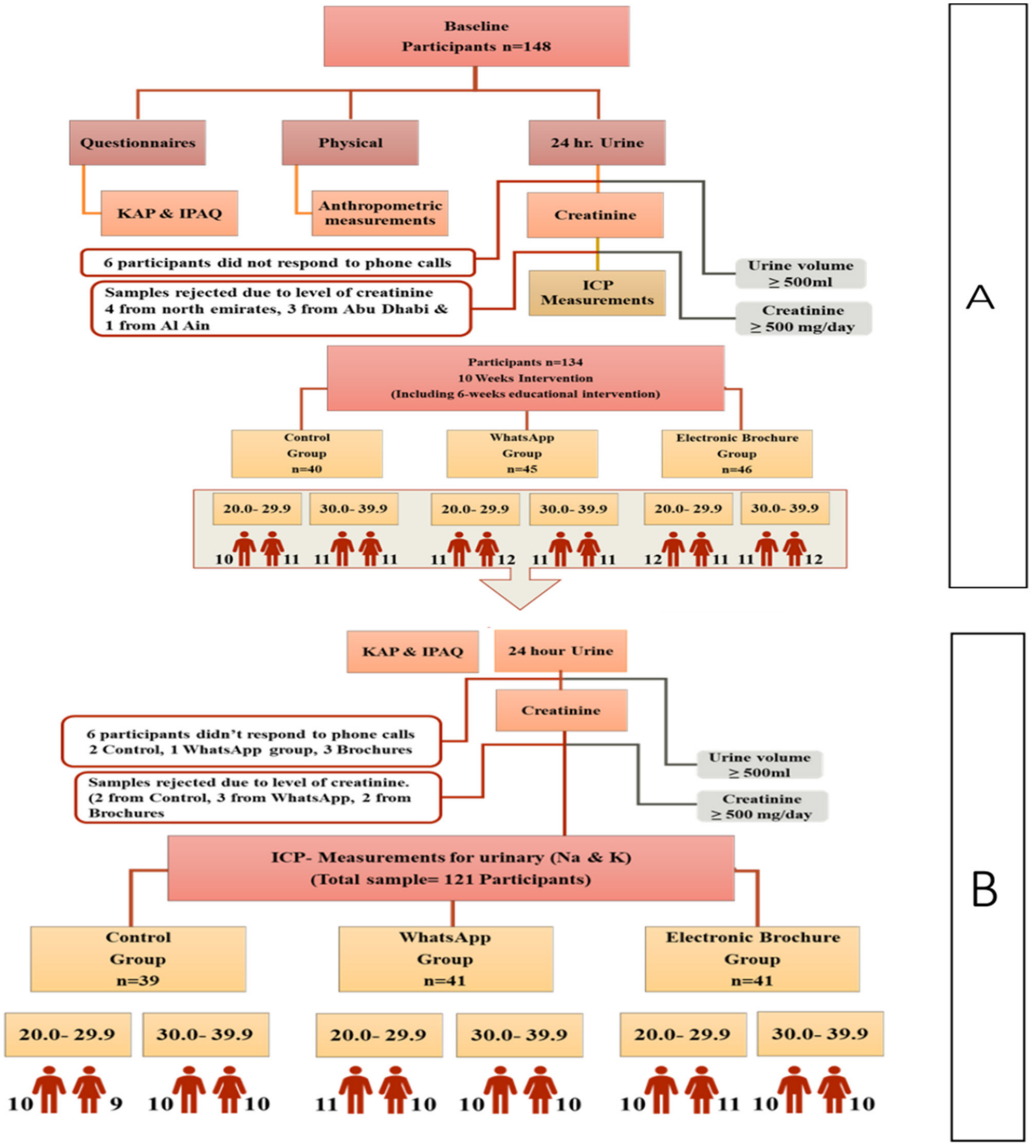
Study flow chart to facilitate salt reduction in UAE using educational materials. KAP, Knowledge, Attitude and Practice; IPAQ, International Physical Activity Questionnaire; ICP, Inductively Coupled Plasma.

A total of 148 participants agreed to participate in the study, out of which 12 participants didn't answer the phones or messages, and 15 participants were excluded due to 24-h urinary creatinine levels below 9-mg/kg body weight for females and < 13 mg/kg body weight for males (4, 16, 17), as shown in Figure 1. At the end of the study, 121 participants completed the study and fulfilled the creatinine inclusion criteria−39 participants were in the control group with female to male ratio (0.95:1.05), while in WhatsApp group, 41 participants completed the study with a female to male ratio of 0.95:1.05 and for in the Electronic brochures group, 41 participants completed the study with a female to male ratio of 1.05:0.95 as shown in Figure 1.

Most of the study population were Emirati (67.8 %). Almost half of the study population were singles and around 75.0% of them were with university degree -educational status. Moreover, more than 50% of them were employed (Table 1).

**Table 1:** Demographic data and 24-h urinary sodium, potassium and creatinine excretions.

**Characteristics**	**Total** **(*n* = 121)** **Mean ± SD^**a**^**	**Female** **(*n* = 60)** **Mean ± SD**	**Male** **(*n* = 61)** **Mean ± SD**	
Age	28.5 ± 5.7	28.3 ± 5.9	28.7 ± 5.5	
BMI	26.0 ± 4.6	25.6 ± 4.2	26.4 ± 4.8	
BMI classification	*n* (%)	*n* (%)	*n* (%)	
Underweight (18.5) (*n*, %)	5 (4.1)	3 (5.0)	2 (3.3)	
Normal weight (18.5–24.9)	49 (40.5)	23 (38.3)	26 (42.6)	
Overweight (25–29.9)	49 (40.5)	25 (41.7)	24 (39.3)	
Obese	18 (14.9)	9 (15.0)	9 (14.8)	
Nationality	*n* (%)	*n* (%)	*n* (%)	
Emirati	82 (67.8)	42 (70.0)	40 (66.7)	
Arabs	39 (32.2)	18 (30.0)	21 (34.4)	
Marital status	*n* (%)	*n* (%)	*n* (%)	
Single	63 (52.1)	23 (38.3)	40 (55.7)	
Married	51 (42.5)	33 (55.0)	19 (31.6)	
Divorced	6.0 (5.0)	4 (6.7)	2 (3.3)	
Educational level	*n* (%)	*n* (%)	*n* (%)	
Undergraduate university level	73 (60.3)	39 (65)	34 (55.7)	
School level (Intermediate or lower)	31 (25.6)	14 (23.3)	17 (27.9)	
Postgraduate level	17 (14.0)	7 (11.7)	10 (16.4)	
Employment status	*n* (%)	*n* (%)	*n* (%)	
Student	29 (24.0)	21 (35.0)	8 (13.1)	
Unemployed	24 (19.8)	18 (30.0)	6 (9.8)	
Employed	68 (56.2)	21(35.0)	47(77.0)	
24-h urinary excretion	Mean ± SD^α^	Mean ± SD	Mean ± SD	*p*-value^c^
Sodium excretion (mg)	3,040.8 ± 665.3^b^	2,836.7 ± 497.3^b^	3,244.8 ± 721^b^	0.001
Salt (mg)	7,601.9 ± 1,635.7^b^	7,091.7 ± 1,243.3^b^	8,112.1 ± 1,822.4^b^	0.001
Potassium excretion (mg)	1,984.4 ± 654.3^d^	1,907.3 ± 608.5^d^	2,061.4 ± 701.5^d^	0.201
Creatinine (mg/day)	1,379.7 ± 339	1,150.2 ± 270.7	1,609.2 ± 226.7	0.001
Creatinine (mg/kg/day)	19.8 ± 4.0	18.1 ± 3.6	21.4 ± 3.8	0.001

α *Data presented as Mean ± standard deviation*.

b *Significantly higher than, the WHO recommendations for sodium and salt, (One- Sample t-test used to compare mean with RDA, Significance at α = 5%.)*.

c Independent-Samples t-test used to assessed Significance at α = 5% for variable according to gender.

d *Significantly lower than, the WHO recommendations for potassium, (One- Sample t-test used to compare mean with RDA, Significance at α = 5%.)*.

### Mean 24-h Urinary Sodium, Potassium, and Creatinine

The mean urinary sodium, salt, and potassium excretion per 24-h for the study population were 3,040.8 ± 665.3, 7,601.9 ± 1,635.7 and 1,984.4 ± 654.3 mg, respectively (Table 1). Moreover, mean urinary excretion for creatinine was 1,379.7 ± 339 mg/day, with significant differences between gender (*p* = 0.001, Table 1). In addition to that, 24-h urinary sodium excretion for total sample and according to gender (*p* = 0.001; Table 1), were significantly higher than WHO recommendation for sodium intake. Moreover, 24-h urinary potassium excretion for total sample and according to gender (*p* = 0.001; Table 1), were lower than WHO recommendation for potassium intake.

In the Control group, there were no significant differences between baseline and endpoint of 10-weeks (6-weeks of educational intervention) for urinary sodium and salt (*p* = 0.055; Table 2). While, participants in the WhatsApp group showed a significant reduction in 24-h urinary sodium and salt after 10-weeks (6 weeks of educational intervention) (3,019.5 ± 593.0 mg vs. 2,741.5 ± 636.0 mg, for sodium; 7,548.7 ± 1,482.5 mg vs. 6,853.6 ± 1,591.0 mg, for salt) (*p* = 0.001; Table 2). In addition to that, participants in the electronic brochures group showed also a significant difference in 24-h urinary sodium excretion and salt between baseline readings and endpoint (*p* = 0.018; Table 2). With respect to 24-h urinary excretion for potassium, WhatsApp group showed a significant improvement in potassium intake compared with baseline readings (*p* = 0.033; Table 3), while the control and Electronic Brochures groups did not show any differences.

**Table 2:** 24-h urinary excretion for sodium, potassium, creatinine, and salt before and after the 6 weeks intervention.

	**Mean** **± SD**^**1**^				
**Variables**	**Stage**	**Control**	**WhatsApp**	**Brochures**	**Total**
		**(*n* = 39)**	**(*n* = 41)**	**(*n* = 41)**	**(*n* = 121)**
Urinary sodium excretion (mg)	Baseline	3,008.7 ± 664.0^α^	3,019.5 ± 593.0^α^	3,094.0 ± 605.0^α^	3,040.8 ± 654.3^α^
	End	3,112.5 ± 609.0^α^	2,741.5 ± 636.0^α^	2,925.2 ± 571.7^α^	2,926.4 ± 664.3^α^
	*p*-value^c^	0.055	0.001	0.018	0.189
Salt (mg/day)	Baseline	7,521.8 ± 1,510.0	7,548.7 ± 1,482.5	7,735.0 ± 1,513.6	7,601.9 ± 1,635.7
	End	7,781 ± 1,622.5	6,853.6 ± 1,591.0	7,313.1 ± 1,429.2	7,316.1 ± 1,626.3
	*p*-value^c^	0.055	0.001	0.018	0.189
Urinary potassium excretion (mg)	Baseline	2,037.0 ± 707^b^	1,868.7 ± 591.2^b^	2,047.0 ± 672.0^b^	1,984.8 ± 658.5^b^
	End	2,014.4 ± 663.0^b^	2,095 ± 630.0^b^	2,063.2 ± 522.4^b^	2,051.5 ± 602.0^b^
	*p*-value^c^	0.867	0.033	0.987	0.409
Sodium to potassium ratio	Baseline	1.5 ± 0.6	1.6 ± 0.8	1.5 ± 0.6	1.5 ± 0.7
	End	1.6 ± 0.7	1.3 ± 0.5	1.4 ± 0.5	1.4 ± 0.6
	*p*-value^c^	0.536	0.009	0.270	0.238
Exceeding WHO recommendation of Sodium intake [*n* (%)] ^d^	Baseline	31 (79.5)	32 (78.0)	31 (75.6)	94 (77.7)
	End	30 (76.9)	27 (65.8)	29 (70.7)	86 (71.1)
	*p*-value^e^	0.323	0.023	0.160	0.004
Urinary creatinine excretion (mg)	Baseline	1,340.6 ± 327.0	1,424.9 ± 371.4	1,369.6 ± 319.0	1,378.4 ± 339
	End	1,324.0 ± 356.0	1,437.7 ± 365.3	1,387.6 ± 304.8	1,383.1 ± 344
	*p*-value^c^	0.369	0.768	0.711	0.902
Creatinine excretion (mg/kg body weight)	Baseline	19.5 ± 3.8	20.1 ± 4.4	19.7 ± 3.14	19.8 ± 4.0
	End	19.0 ± 3.6	20.7 ± 4.2	20.0 ± 3.4	19.9 ± 4.2
	*p*-value^c^	0.651	0.780	0.635	0.908

1 *Data presented as Mean ± standard deviation*.

α *Significantly higher than, the WHO recommendations for sodium, (One- Sample t-test used to compare mean with RDA = 2,300 mg/day, Significance at α = 5%.)*.

b *Significantly lower than, the WHO recommendations for potassium, (One- Sample t-test used to compare mean with RDA = 3,510 mg/day, Significance at α = 5%.)*.

c *Paired t-test according to time of measurements (Baseline vs. endpoint) within the same group*.

d *Data presented as number and frequency*.

e *Paired t-test according to time of measurements (Baseline vs. endpoint) within the same group, each reading above than WHO recommendation earned 1, while the other zero, to estimate mean and perform paired t-test*.

**Table 3:** Salt-related attitudes among study population by intervention groups before and after the 6 weeks intervention.

**Variable1**	**Stage**	**Control**	**WhatsApp**	**Brochure**
		**(*n*, %)**	**n (*n*, %)**	**(*n*, %)**
How much salt do you think you consume (Just the right amount)	Baseline *n* (%)^α^	24 (61.5)	27 (65.8)	24 (58.5)
	Mean ± SD^b^	0.62 ± 0.51	0.66 ± 0.43	0.59 ± 0.40
	Endpoint *n* (%)	25 (64.1)	31 (70.6)	27 (65.8)
	Mean ± SD	0.64 ± 0.42	0.71 ± 0.35	0.66 ± 0.33
	*p*-value^c^	0.323	0.133	0.099
Are you concerned about the amount of salt/sodium in the diet (Yes)	Baseline *n* (%)	6 (15.4)	4 (9.7)	5 (12.2)
	Mean ± SD	0.15 ± 0.49	0.01 ± 0.45	0.12 ± 0.48
	Endpoint *n* (%)	6 (15.4)	9 (22.0)	7 (17.1)
	Mean ± SD	0.15 ± 0.49	0.22 ± 0.32	0.17 ± 0.46
	*p*-value	1.00	0.012	0.160
Reducing added salt to foods is important to you (Agree)	Baseline *n* (%)	10 (25.6)	9 (22.0)	10 (24.4)
	Mean ± SD	0.26 ± 0.35	0.22 ± 0.30	0.24 ± 0.31
	Endpoint *n* (%)	11 (28.2)	22 (53.7)	29 (70.7)
	Mean ± SD	0.28 ± 0.32	0.54 ± 0.42	0.71 ± 0.33
	*p*-value	0.323	0.001	0.001
Reducing consumption of processed foods is important to you (Agree)	Baseline *n* (%)	15 (38.5)	10 (24.4)	13 (31.7)
	Mean ± SD	0.39 ± 0.47	0.24 ± 0.31	0.32 ± 0.41
	Endpoint *n* (%)	14 (35.9)	28 (68.3)	30 (73.1)
	Mean ± SD	0.36 ± 0.49	0.68 ± 0.48	0.73 ± 0.36
	*p*-value	0.802	0.001	0.001
Reducing your sodium intake is important to you (Agree)	Baseline *n* (%)	12 (30.8)	10 (24.4)	14 (34.1)
	Mean ± SD	0.31 ± 0.36	0.24 ± 0.31	0.34 ± 0.39
	Endpoint *n* (%)	14 (35.9)	25 (61.0)	19 (46.3)
	Mean ± SD	0.36 ± 0.30	0.61 ± 0.38	0.46 ± 0.42
	*p*-value	0.785	0.001	0.012

*^1^Attitude was assessed based on a three-point Likert scale but only answers of “agree” are presented, each agree answer earned 1 score, while the other zero, to estimate mean and perform paired t-test*.

α *Data presented as number and frequency*.

b *Data presented as Mean ± standard deviation*.

c *Paired t-test according to time of measurements (Baseline vs. endpoint) within the same group*.

Moreover, a 2 × 3 Mixed repeated measures ANOVA (time vs. group) revealed a significant main effect for time on 24-h urinary sodium excretion [*F*_(1, 117)_ = 11.812; *p*= 0.001; $\eta_{p}^{2}=$ 0.092], and for interaction between time and group [*F*_(2, 117)_ = 10.093; *p* < 0.001; $\eta_{p}^{2}$ = 0.147], No significant effect was detected for group [*F*_(2, 117)_ = 0.590; *p* = 0.556; $\eta_{p}^{2}$ = 0.010].

There were no significant differences between baseline and endpoint after 6-weeks of education for 24-h urinary excretion for creatinine and creatinine as mg/kg body weight for the Control group and the two intervention groups (Table 2).

The percentage of participants exceeding WHO recommendation of Sodium intake for total population was 77.7% at baseline and became 71.1% at the end of the intervention period, with a significant reduction (*p* = 0.004, Table 2). In the WhatsApp group, the percentage of participants exceeding WHO recommendation of sodium intake was significantly lower after intervention (*p* = 0.023). While for the electronic brochure, there was no significant change (*p* = 0.160, Table 2).

### Salt-Related Knowledge, Attitudes and Practices

Although 77.7% of the study population exceeded WHO recommendation of sodium intake according to 24-h urinary excretion, around 60.0% of the study population thought they just consumed the right amount at baseline (Table 3). After 6-weeks of intervention, 71.1% of the study population exceeded WHO recommendation of sodium intake according to 24-h urinary excretion, while around 65% believed that they just consumed the right amount of salt according to the KAP questionnaire response. This indicated that the study population is not able to give the right judgment of correct estimation of salt intake (Table 3). With respect to the attitude of the study population, the WhatsApp group was significantly more concerned (*p* = 0.028) about the amount of salt intake after 10-weeks (6-weeks of educational intervention) compared with baseline (Table 3).

After educational intervention, both WhatsApp and Electronic Brochures groups showed a significant increase in awareness on the importance of reducing salt intake compared with baseline readings (*p* = 0.001), as shown in Table 3. Moreover, there was a significant increase in the attitude toward agreeing on the importance of reducing consumption of processed foods and reducing sodium intake after educational intervention for both WhatsApp and Electronic Brochures (*p* < 0.05).

### Salt-Related Practices Among Study Population

After 10-weeks (6-weeks of educational intervention), the intervention groups (WhatsApp and Electronic Brochures) showed a significant increase (*p* < 0.05), in the percentage of participants checking food labels often (Table 4). In addition to that, there was a significant increase (*p* < 0.05) in checking labels specifically for salt/sodium content (often) and more participants reported that salt/sodium content on the label affects purchasing decisions (often) (*p* < 0.05; Table 4). However, the WhatsApp intervention group significantly improved the practice of buying “low salt” foods (*p* = 0.002) as shown in Table 4.

**Table 4:** Salt-related practices among study population by intervention groups (*n* = 121) before and after the 6 weeks intervention, check supplementary.

**Variable1**	**Stage**	**Control (Mean)**	**WhatsApp**	**Brochure**
			**(*n*, %)**	**(*n*, %)**
Check food labels (Often)	Baseline *n* (%)^α^	7 (17.9)	9 (22.0)	10 (24.4)
	Mean ± SD^b^	0.18 ± 0.33	0.22 ± 0.42	0.24 ± 0.43
	Endpoint *n* (%)	8 (20.0)	20 (48.8)	14 (34.1)
	Mean ± SD	0.20 ± 0.36	0.49 ± 0.51	0.34 ± 0.38
	*p*-value^c^	0.323	0.003	0.044
Information on food labels affects purchasing decisions (Often)	Baseline *n* (%)	5 (12.8)	7 (17.5)	7 (17.5)
	Mean ± SD	0.13 ± 0.35	0.18 ± 0.37	0.18 ± 0.37
	Endpoint *n* (%)	5 (12.8)	17 (41.5)	14 (34.1)
	Mean ± SD	0.13 ± 0.35	0.42 ± 0.50	0.38 ± 0.48
	*p*-value	1.00	0.001	0.006
Check labels specifically for salt/sodium content (Often)	Baseline *n* (%)	2 (5.1)	4 (9.8)	2 (4.9)
	Mean ± SD	0.05 ± 0.22	0.1 ± 0.28	0.05 ± 0.22
	Endpoint *n* (%)	3 (7.0)	18 (45.0)	18(45.0)
	Mean ± SD	0.08 ± 0.26	0.45 ± 0.50	0.45 ± 0.50
	*p*-value	0.323	0.001	0.001
Salt/sodium content on label affects purchasing decisions (Often)	Baseline *n* (%)	3 (7.5)	3 (7.3)	2 (5.0)
	Mean ± SD	0.08 ± 0.26	0.07 ± 0.26	0.05 ± 0.22
	Endpoint *n* (%)	3 (7.5)	13 (31.7)	9 (22.5)
	Mean ± SD	0.08 ± 0.26	0.32 ± 0.47	0.23 ± 0.40
	*p*-value	1.00	0.001	0.012
Try to buy “low salt” foods (Often)	Baseline *n* (%)	1 (2.6)	1 (2.4)	2 (4.9)
	Mean ± SD	0.03 ± 0.16	0.02 ± 0.16	0.05 ± 0.02
	Endpoint *n* (%)	1 (2.6)	10 (24.4)	4 (9.8)
	Mean ± SD	0.03 ± 0.16	0.24 ± 0.42	0.10 ± 0.30
	*p*-value	1.00	0.002	0.160
Add salt to food during cooking (Often)	Baseline *n* (%)	31 (79.5)	28 (68.3)	30 (73.1)
	Mean ± SD	0.80 ± 0.40	0.68 ± 0.46	0.73 ± 0.44
	Endpoint *n* (%)	32 (82.1)	15 (36.6)	18 (43.9)
	Mean ± SD	0.82 ± 0.43	0.36 ± 0.49	0.44 ± 0.50
	*p*-value	0.323	0.001	0.001
Use Stock Cubes during cooking (Often)	Baseline *n* (%)	16 (41.0)	14 (34.1)	11 (26.8)
	Mean ± SD	041 ± 0.50	0.34 ± 0.48	0.27 ± 0.45
	Endpoint *n* (%)	17 (43.6)	5 (12.2)	5 (12.2)
	Mean ± SD	0.44 ± 0.50	0.12 ± 0.33	0.12 ± 0.33
	*p*-value	0.323	0.002	0.012
Add salt to food at the table (Often)	Baseline *n* (%)	9 (23.1)	7 (17.1)	7 (17.5)
	Mean ± SD	0.23 ± 0.42	0.17 ± 0.38	0.17 ± 0.38
	Endpoint *n* (%)	11 (27.5)	1 (2.4)	1 (2.4)
	Mean ± SD	0.28 ± 0.45	0.02 ± 0.15	0.02 ± 0.15
	*p*-value	0.160	0.012	0.012
Add salt before tasting the food (Often)	Baseline *n* (%)	13 (33.3)	8 (19.5)	9 (22.0)
	Mean ± SD	0.33 ± 0.47	0.20 ± 0.40	0.22 ± 0.42
	Endpoint *n* (%)	11 (27.5)	0 (0.0)	1 (2.4)
	Mean ± SD	0.28 ± 0.45	0.00 ± 0.00	0.02 ± 0.15
	*p*-value	0.160	0.003	0.003
Did you try to use spices to reduce salt (Yes)	Baseline *n* (%)	14 (35.9)	12 (29.3)	16 (39.0)
	Mean ± SD	0.36 ± 0.48	0.29 ± 0.46	0.39 ± 0.46
	Endpoint *n* (%)	13 (33.3)	31 (75.6)	29 (70.7)
	Mean ± SD	0.33 ± 0.47	0.76 ± 0.42	0.71 ± 0.45
	*p*-value	0.323	0.001	0.001

*^1^ Answer options for practice questions included often, sometimes, and never. Only answers of often are presented, each often answer earn 1 score*.

α *Data presented as number and frequency*.

b *Data presented as Mean ± standard deviation*.

c *Paired t-test according to time of measurements (Baseline vs. endpoint) within the same group*.

Practices toward adding salt during cooking, at the table, before tasting foods and using Stock Cubes during cooking were significantly reduced with (*p* < 0.05), in both intervention groups (WhatsApp and Electronic Brochures) after educational intervention (Table 4). Moreover, there was a significant increase in using spices, herbs and lemons as alternative of salt in both intervention groups (WhatsApp and Electronic Brochures) after 6-weeks of educational intervention (Table 4).

### Food and Health Related Knowledge Among Study Population

Food related knowledge was significantly improved for both the WhatsApp and Electronic Brochure intervention groups after 10-weeks (6-weeks of educational intervention) with values of 3.5 ± 2.8 and 3.7 ± 2.5 out of 10, respectively for both groups at baseline. However, after 10-weeks (6-weeks of educational intervention), food related knowledge become; 8.5 ± 1.9 and 8.9 ± 1.6 out of 10 for both the WhatsApp and Electronic Brochure, respectively (*p* = 0.001) as shown in Table 5.

**Table 5:** Food and Health related knowledge for the study population (*n* = 121), before and after the 6 weeks intervention.

**Variables**		**Control** **(*n* = 39) Mean ± SD^**α**^**	**Classification^**c**^**	**WhatsApp (*n* = 41) Mean ± SD**	**Classification**	**Brochures (*n* = 41) Mean ± SD**	**Classification**
Food related knowledge	Baseline	3.8 ± 1.8	Poor	3.5 ± 2.8	Poor	3.7 ± 2.5	Poor
	Endpoint	4.4 ± 1.5	Poor	8.5 ± 1.9	Good	8.9 ± 1.6	Good
	*p*-value^b^	0.142		0.001		0.001	
Medical related knowledge	Baseline	6.9 ± 1.9	Fair	6.4 ± 3.0	Fair	6.7 ± 2.7	Fair
	Endpoint	7.0 ± 2.0	Fair	9.3 ± 1.3	Good	8.9 ± 1.4	Good
	*p*-value	0.604		0.001		0.001	

α *Data presented as Mean ± standard deviation*.

b *paired t-test according to time of measurements (Baseline vs. endpoint) within the same group*.

c *Score percentage for Knowledge < 60 classified as “Poor”, 60–70 classified as “Fair”, >70 classified as “Good” (20)*.

Health related knowledge for the study population was significantly improved after 10-weeks (6-weeks of educational intervention) for both WhatsApp and Electronic Brochures groups with (*p*-value = 0.001), as shown in Table 5.

Most of the study population failed to identify food rich in sodium, about 20 to 26% were able to recognize Arabic and Iranian bread as high sodium sources at baseline (Table 6). However, after 6-weeks of educational intervention, 80–95% of participants in the WhatsApp and Electronic Brochures groups were able to recognize Arabic and Iranian bread as high sodium sources (*p* = 0.001) as shown in Table 6. About 40 to 70% of study population at baseline were able to recognize canned vegetables, cheddar cheese, pickles, salad dressing oil, Ketchup, tomato paste, chicken cubes, and Instant noodle as rich food sources of sodium (Table 6). This percentage increased significantly up to 95% and above for intervention group after educational intervention as shown in Table 6 (*p* = 0.001).

**Table 6:** The number and percentage of participants who correctly answered food related knowledge, before and after the 6 weeks intervention, check supplementary.

**Sodium content in the following foods is1**	**Stage**	**Control** **(*n* = 39)**	**WhatsApp** **(*n* = 41)**	**Brochure** **(*n* = 41)**
Arabic bread (High)	Baseline *n (%*)^α^ Mean ± SD^b^	10 (25.6) 0.26 ± 0.43	10 (24.3) 0.24 ± 0.43	8 (19.5) 0.20 ± 0.40
	Endpoint *n (%*) Mean ± SD	11 (28.2) 0.28 ± 0.45	34 (82.9) 0.83 ± 0.46	38 (95.0) 0.95 ± 0.22
	*p*-value^c^	0.323	0.001	0.001
Iranian bread (High)	Baseline *n (%*) Mean ± SD	12 (30.8) 0.31 ± 0.36	13 (31.7) 0.32 ± 0.47	14 (34.1) 0.34 ± 0.48
	Endpoint *n (%*) Mean ± SD	13 (33.3) 0.33 ± 0.47	37 (92.5) 0.93 ± 0.27	36 (90.0) 0.90 ± 0.26
	*p*-value	0.323	0.001	0.001
Rice- Egyptian (Low)	Baseline *n (%*) Mean ± SD	21 (53.8) 0.54 ± 0.49	20 (48.8) 0.49 ± 0.50	22 (53.7) 0.54 ± 0.50
	Endpoint *n (%*) Mean ± SD	23 (0.59) 0.59 ± 0.50	24 (58.5) 0.59 ± 0.49	35 (85.4) 0.85 ± 0.33
	*p*-value	0.160	0.044	0.001
Rice-Basmati (Low)	Baseline *n (%*) Mean ± SD	19 (48.7) 0.49 ± 0.50	22 (53.7) 0.54 ± 0.50	20 (48.8) 0.49 ± 0.50
	Endpoint *n (%*) Mean ± SD	21 (53.8) 0.54 ± 0.50	31 (75.6) 0.76 ± 0.42	21 (51.2) 0.51 ± 0.40
	*p*-value	0.160	0.002	0.323
Milk/yogurt (Low)	Baseline *n (%*) Mean ± SD	19 (48.7) 0.49 ± 0.50	18 (43.9) 0.44 ± 0.50	17 (41.4) 0.41 ± 0.50
	Endpoint *n (%*) Mean ± SD	18 (43.9) 0.44 ± 0.50	30 (73.1) 0.73 ± 0.43	35 (85.4) 0.85 ± 0.33
	*p*-value	0.323	0.001	0.001
Fresh red meat (Low)	Baseline *n (%*) Mean ± SD	13 (33.3) 0.33 ± 0.47	11 (26.8) 0.27 ± 0.45	13 (31.7) 0.32 ± 0.47
	Endpoint *n (%*) Mean ± SD	15 (38.5) 0.39 ± 0.49	21 (51.2) 0.51 ± 0.50	33 (80.5) 0.85 ± 0.38
	*p*-value	0.160	0.001	0.001
Fresh poultry (Low)	Baseline *n (%*) Mean ± SD	15 (38.5) 0.39 ± 0.49	11 (26.8) 0.27 ± 0.45	12 (29.3) 0.29 ± 0.46
	Endpoint *n (%*) Mean ± SD	14 (35.8) 0.36 ± 0.38	21 (51.2) 0.51 ± 0.50	20 (48.8) 0.49 ± 0.50
	*p*-value	0.3232	0.001	0.003
Fruits (Low)	Baseline *n (%*) Mean ± SD	25 (64.1) 0.64 ± 0.49	23 (57.5) 0.58 ± 0.50	24 (58.5) 0.59 ± 0.49
	Endpoint *n (%*) Mean ± SD	22 (53.7) 0.54 ± 0.50	37 (92.5) 0.93 ± 0.27	38 (95.0) 0.95 ± 0.22
	*p*-value	0.083	0.001	0.001
Fresh vegetables (Low)	Baseline *n (%*) Mean ± SD	26 (66.6) 0.67 ± 0.49	28 (68.3) 0.68 ± 0.46	26 (63.4) 0.63 ± 0.48
	Endpoint *n (%*) Mean ± SD	24 (61.5) 0.62 ± 0.48	41 (100) 100 ± 0.00	39 (95.1) 0.95 ± 0.16
	*p*-value	0.160	0.001	0.001
Canned vegetables (High)	Baseline *n (%*) Mean ± SD	28 (71.8) 0. 72 ± 0.46	24 (58.5) 0.59 ± 0.50	28 (68.3) 0.68 ± 0.46
	Endpoint *n (%*) Mean ± SD	27 (69.2) 0.69 ± 0.47	37 (92.5) 0.93 ± 0.27	39 (95.1) 0.95 ± 0.16
	*p*-value	0.323	0.001	0.001
Cheddar cheese (High)	Baseline *n (%*) Mean ± SD	29 (74.4) 0.74 ± 0.45	27 (65.9) 0.66 ± 0.47	28 (68.3) 0.68 ± 0.46
	Endpoint *n (%*) Mean ± SD	28 (71.8) 0. 72 ± 0.46	39 (95.1) 0.95 ± 0.16	39 (95.1) 0.95 ± 0.16
	*p*-value	0.323	0.001	0.001
Pickles (High)	Baseline *n (%*) Mean ± SD	28 (71.8) 0. 72 ± 0.46	30 (73.1) 0.73 ± 0.43	28 (68.3) 0.68 ± 0.46
	Endpoint *n (%*) Mean ± SD	33 (84.6) 0.85 ± 0.38	39 (95.1) 0.95 ± 0.16	38 (95.0) 0.95 ± 0.22
	*p*-value	0.023	0.002	0.001
Olive oil (Low)	Baseline *n (%*) Mean ± SD	18 (43.9) 0.44 ± 0.50	20 (48.8) 0.49 ± 0.50	16 (39.0) 0.39 ± 0.49
	Endpoint *n (%*) Mean ± SD	22 (53.7) 0.54 ± 0.50	35 (85.4) 0.85 ± 0.33	24 (58.5) 0.59 ± 0.49
	*p*-value	0.044	0.001	0.003
Salad dressing oil (High)	Baseline *n (%*) Mean ± SD	25 (64.1) 0.64 ± 0.49	24 (58.5) 0.59 ± 0.49	22 (53.7) 0.54 ± 0.50
	Endpoint *n (%*) Mean ± SD	26 (66.6) 0.67 ± 0.49	38 (95.0) 0.95 ± 0.22	37 (92.5) 0.93 ± 0.27
	*p*-value	0.323	0.001	0.001
Ketchup (High)	Baseline *n (%*) Mean ± SD	21 (53.8) 0.54 ± 0.49	20 (48.8) 0.49 ± 0.50	22 (53.7) 0.54 ± 0.50
	Endpoint *n (%*) Mean ± SD	23 (0.59) 0.59 ± 0.50	39 (95.1) 0.95 ± 0.16	39 (95.1) 0.95 ± 0.16
	*p*-value	0.160	0.001	0.001
Tomato paste (High)	Baseline *n (%*) Mean ± SD	21 (53.8) 0.54 ± 0.49	22 (53.7) 0.54 ± 0.50	21 (51.2) 0.51 ± 0.50
	Endpoint *n (%*) Mean ± SD	22 (53.7) 0.54 ± 0.50	39 (95.1) 0.95 ± 0.16	38 (95.0) 0.95 ± 0.22
	*p*-value	0.323	0.001	0.001
Corn flakes (High)	Baseline *n (%*) Mean ± SD	8 (20.5) 0.21 ± 0.40	8 (19.5) 0.20 ± 0.40	10(24.4) 0.24 ± 0.43
	Endpoint *n (%*) Mean ± SD	7 (17.9) 0.18 ± 0.38	33 (80.5) 0.81 ± 0.38	35 (85.4) 0.85 ± 0.33
	*p*-value	0.323	0.001	0.001
Chicken cubes (High)	Baseline *n (%*) Mean ± SD	29 (74.4) 0.74 ± 0.45	26 (63.4) 0.63 ± 0.48	28 (68.3) 0.68 ± 0.46
	Endpoint *n (%*) Mean ± SD	31 (79.5) 0.80 ± 0.42	39 (95.1) 0.95 ± 0.16	40 (97.6) 0.98 ± 0.14
	*p*-value	0.160	0.001	0.001
Instant noodle (High)	Baseline *n (%*) Mean ± SD	27 (69.2) 0.69 ± 0.47	29 (70.7) 0.71 ± 0.45	28 (68.3) 0.68 ± 0.46
	Endpoint *n (%*) Mean ± SD	26 (66.6) 0.67 ± 0.49	38 (95.0) 0.95 ± 0.22	39 (95.1) 0.95 ± 0.16
	*p*-value	0.323	0.002	0.001

^1^ *The correct answers are provided in brackets next to each variable. Each correct answer earned 1 score*.

α *Data presented as number and percentage*.

b *Data presented as Mean ± standard deviation*.

c *Paired t-test according to time of measurements (Baseline vs. endpoint) within the same group*.

After 6-weeks of educational intervention using WhatsApp and Electronic Brochures, the majority of the study population were able to recognize corn takes as a rich source of sodium (*p* = 0.001) (Table 6). At the end of Intervention, the WhatsApp group showed a significant difference compared with baseline for 19 food items out of 19 with 100% improvement compared with baseline. Similarly, the Electronic Brochures group showed a significant difference compared with baseline for 18 food items out of 19 with 94.7% improvement. The Control group also showed a significant improvement in recognizing pickle foods as a high salt food at the endpoint after 6-weeks (*p* < 0.05) (Table 6). The Control group showed improvement in 1 food item compared with 19, with 5.3% improvement at the end of intervention.

### Physical Activity of the Study Population Before and After Intervention

Physical activity of the study population was measured using 7-days self-reported short IPAQ for the three groups at baseline and intervention, showed no significant differences at baseline and after 10-weeks (6-weeks of educational intervention), as shown in Table 7.

**Table 7:** Physical activity for study population before and after the 6 weeks intervention using IPAQ questionnaire.

	**Control (*****n*** **=** **39)** **Mean** **±SE^α^**		**WhatsApp (*****n*** **=** **41)** **Mean** **±SE**		**Brochures (*****n*** **=** **41)** **Mean** **±SE**	
	**Baseline**	**Endpoint**	**Baseline**	**Endpoint**	**Baseline**	**Endpoint**
Low intensity (min/week)	205.8 ± 25.4	195.6 ± 26.6	198.0 ± 20.3	176.5 ± 17.1	181.4 ± 16.4	191.8 ± 20.2
*p*-value^b^	0.692		0.482		0.398	
Moderate intensity (min/week)	87.7 ± 13.1	79.0 ± 16.0	90.0 ± 16.5	103.7 ± 17.3	86.7 ± 9.5	82.3 ± 9.1
*p*-value^b^	0.679		0.476		0.743	
High intensity (min/week)	54.1 ± 12.5	41.1 ± 7.3	54.9 ± 7.1	48.8 ± 6.4	43.3 ± 6.3	47.1 ± 6.6
*p*-value^b^	0.369		0.414		0.415	

α *Data presented as Mean ± Standard error*.

b *Independent-Samples t-test used to assessed Significance at p < 0.05 according to gender*.

## Discussion

To our knowledge, this is the first study in the UAE, GCC, and Arab Countries, aimed to measure the effectiveness of educational materials using social media approaches such as WhatsApp and Electronic Brochures for 6-weeks with 24-h urinary sodium excretion levels to facilitate salt reduction. Out of 148 participants, 121 participants completed the study according to urine volume and creatinine levels based on the following studies (4, 15, 16, 21–25).

The urinary sodium excretion in the current study was significantly higher than the WHO recommendations, which is (2,300 mg/day) and significantly lower for potassium (3,150 mg/day). The result of the current study showed an increase in salt consumption by 12% in a sample of UAE population compared with the study conducted in 2015, which showed level of salt intake around 6,783.5 mg (4). It is worth noting that the age group in the current study is up to 40 years, while Jarrar et al. study was up to 60 years old. The highest urinary sodium excretion was reported in age groups 20–29.9 and 30–39.9 years old compared with age groups 40–49.9 years and above and 50 years old and above (4). Therefore, the difference in the age group may explain the differences in salt intake between the current study compared with the previous study. Nevertheless, the common result between the three studies is that the mean urinary sodium excretion indicates higher than the WHO recommendation for salt intake.

There are several salt reduction strategies or approaches all over the world, these strategies were implemented as one strategy or a combination of two or more strategies or as comprehensive strategies (2, 26, 27). These reduction strategies or approaches for salt intakes may include one or more of the following such as educational approaches (individual/ group). Generally, most of the intervention's strategies are based on education or counseling lasting for 2 weeks to <12 months (26, 28–30). The duration of the intervention varies according to the aim of the study, some interventions lasted for 2 weeks (28), or 6-weeks as reported by (30). Others have used smartphone apps as tools for educational intervention and behavioral change for 4-weeks (29, 31, 32). Some studies have also used short SMS messages and pamphlets as tools to deliver educational materials for 30-days (33, 34), or 3 months (30).

In the current study, after 10-weeks (6-weeks of educational intervention), the intervention groups (WhatsApp and Electronic Brochure) showed a significant reduction in salt intake compared with baseline. In the WhatsApp Intervention group, the reduction was 695.1 mg (~0.7 g) with −9.2% compared with baseline, and the reduction in salt intake for Electronic Brochure group was 421.9 mg (~0.4 g) with (−5.4%). Many other studies that used educational approach as intervention tool showed similar results. Eyles et al. used smartphone application (app) as an intervention tool for 4 weeks. The app tool, resulted in a significant reduction in salt (~0.7 g) compared with baseline (29).

Another study conducted using Smartphone technology showed a significant reduction in salt intake after 4-weeks of intervention with (~0.8 g) (31).

Anderson et al. (10) reported a reduction of 1.2 g/day in 24-h urinary sodium excretion for Intervention group using multicomponent-behavioral interventions for 6 months. Moreover, He et al. conducted a cluster randomized clinical trial to determine whether an education program delivered to primary school children as part of the usual curriculum could lower salt intake in children and their families (35). The mean salt intake reduction as measured by urinary excretion in children was 1.9 g/day while for families it was −2.9 g/day.

Many studies using education/counseling approach reported a reduction in salt intake with range various from 0.4 to 2.9 g/day (10, 31, 35–37). However, many studies reported the commitment toward salt reduction rely mainly on the educations of the participants and updating or maintaining education to maintain good salt reduction intake (31, 35).

Moreover, Intervention approaches/ strategies using three approaches such as education, reformulation and taxes showed a significant reduction in salt intakes of ~4 g/day as reported in Finland and Japan, 3 g/day in Turkey and 1.3 g/day in the UK (26).

In the current study, the reduction in salt intake in the intervention groups was associated with improvement in food and health related knowledge. In food related knowledge, the WhatsApp group showed 2.4 times improvements in scores compared with baseline after 10-weeks (6-weeks of educational intervention), while the Electronic Brochure group showed 2 times improvement compared with baseline.

The health-related knowledge improved by 1.4 times in the intervention groups compared with baseline. Moreover, this improvement in food and health related knowledge reflected in an improvement in the attitudes and practices toward the selection and purchasing of low salt foods. Although this improvement increased the recognition of bread as a high source of salt, the intake of Arabic bread was reduced only by 4% which is the lowest compared with other food items that reached 10 to 30% reduction. Pickled foods showed the highest reduction after 10-weeks (6-weeks of educational intervention) with almost 32% reduction, followed by chicken cubes with 22% reduction. Moreover, Instant Indomi (Instant noodles) showed a reduction of 20%, cheeses intake was reduced by 10–20% while Tomato paste showed only 8% reduction after 10-weeks (6-weeks of educational intervention).

Several studies conducted in Arab Gulf Countries such as UAE, Qatar and Kuwait (4, 37), showed that bread and bakery products are considered as staple food in UAE, Arab Gulf Countries, Arab World and most of the Middle East countries (4, 38, 39). It is not easy to reduce the intake of bread and other baked products even if it is one of the main sources of salt in the diet. However, reduction of salt content in bread may can contribute to reduce salt intake (38).

A study conducted in Jordan aimed to measure salt contents of Arabic bread. Sixty-eight bread samples were collected from 13 different bakeries from Amman, mean salt content was 1.19 ± 0.21 g /100 g bread, while other local bread “Shrak” was 2.06 ± 0.19 1.19 ± 0.21 g /100 g (39). Therefore, one of the best approaches to reduce salt intake is to provide low salt bread or place a regulation standard to control salt in the bread. Emirates Authority for Standardization & Metrology (ESMA), initiative for voluntary salt reduction in bread began in February 2020 and it will be obligatory on 2022 (40), this aimed to reduce 50% of salt content in the bread “Arabic” to be 500 g salt/100 g of bread.

Thus, make bread among main contributing sources of high salt intake in UAE similar to Kuwait, Jordan and Qatar (38, 39).

Educational intervention improved significantly the identification of foods with low sodium contents such as fresh fruits and vegetables, rice, yogurt, and fresh meat. The improvement was also reported significantly in the attitude of the intervention group. Reducing added salt and low consumption of processed foods become significantly important for the intervention groups compared with the control group after 6-weeks of educational intervention.

The improvement in knowledge and attitude contributed to improvement in practice of the intervention groups. The intervention group showed significant improvement in the practice part of the KAP questionnaire. Checking food labels specifically for salt/sodium content affected purchasing decisions. In addition to that, an increased percentage of participants tried to buy “low salt” and “no added salt” foods in the intervention groups compared with the control group.

Moreover, addition of salt during cooking reduced significantly in the intervention groups and the use of chicken cubes reduced whilst the use of salt alternatives increased significantly, in addition to significant reduction in adding table salt and adding salt before tasting foods. This improvement in the KAP components in the intervention group was translated into reduction in salt intake by the intervention groups in the food frequency questionnaire, which reported a significant reduction in using Table salt by 13% and chicken cubes by almost 20% compared with baseline.

The intervention showed an increase in the use of spices and herbs as alternatives for salt. Therefore, the current study showed a positive relationship between KAP toward salt intake and reducing salt intake. Haron et al. (41) reported a positive correlation between high knowledge scores with more controlled blood pressure compared to those who scored less.

Multiple approach was applied in countries as a multi-component programs (consumer awareness campaign, increased availability of low-sodium foods at school, worksite and restaurants, voluntary reformulation of processed foods to lower the sodium content) (7, 32). This approach showed a positive effect in salt reduction by 15% in UK (6) and 24% reduction in South Korean (7).

The 24-h urinary potassium excretion is correlated with dietary potassium intake (23, 42). In the current study all participants failed to achieve WHO recommendations for potassium intake. In addition to that, high urinary sodium to potassium ratio can be an indicator of the need for reducing sodium intake and increasing potassium intake (14). Therefore, WHO has suggested sodium: potassium ratio of ~1.00, which will be associated with low risk for development of CVDs (43). Although the WhatsApp group showed a significant improvement in the sodium to potassium ratio, the ratio was still above one and participants failed to achieve the recommended intake of potassium. This means promoting, the intake of foods rich in potassium is just as important as reducing the intake of foods rich in sodium/salt.

The current study proved that improving KAP through providing educational materials was associated with lowering salt intake in a sample of UAE population. Moreover, the digital platform approach such as WhatsApp and Electronic Brochure were effective in terms of salt reduction. This is considered the first step or one single element of a comprehensive approach starting with educational materials, which aimed to facilitate consumer awareness regarding low and high salt foods. In addition to reformulation of staple foods (particularly bread) to reduce salt content, building a good environment for the food industry to be part of this initiative to reduce salt intake is important in UAE and in Arab Gulf Countries, and middle east region. In addition to that, including the study population in proper decision making regarding best approaches and tools to reduce salt intake is also important to make the process of salt reduction more visible and applicable.

Despite the significant findings of this study, it could be of a better value if there is more than one follow-up to the study to measure the study population's responses toward salt reduction for longer period. Moreover, measuring salt reduction along with blood pressure will give better responses toward blood pressure in UAE. In addition to that, for the KAP part of the study, including more participants in term of age groups and number will be give more useful results.

## Conclusion

This study proven that the UAE population respond positively to salt reduction initiative using digital platform (WhatsApp and Electronic brochures) to deliver educational materials for 6-weeks. WhatsApp group showed a significant reduction in salt intake after intervention, compared with Electronic brochures and Control groups. Moreover, this reduction in salt intake was associated with significant reduction with 10% of the participants who exceeds WHO recommendation for sodium intake for WhatsApp group. KAP toward salt intake was improved for intervention group at the end of the study. In addition to that, there was a significant reduction in the practice toward adding salt during cooking, use of table salt, and adding salt before tasting. These results indicate the study population of UAE is ready to change the consumption and practice toward salt intake. In order, to make it more durable it is requiring the industry and the policy makers in UAE to provide low salt foods/products and encourage reformulation of some staple foods such as bakery products particularly bread.

This is the first intervention study conducted in UAE, that used digital platform to reduce salt intake using KAP questionnaire, and 24-h urinary sodium, potassium and creatinine excretion as a tool for measurement.

## Data Availability Statement

The raw data supporting the conclusions of this article will be made available by the authors, without undue reservation.

## Ethics Statement

The studies involving human participants were reviewed and approved by University Research Ethics Committee at Oxford Brookes University (UREC), Number: 191337. In addition to, ethical approval from United Arab Emirates University (UAEU) Research Ethics Committee (ERS_2020_6107). The patients/participants provided their written informed consent to participate in this study. Written informed consent was obtained from the individual(s) for the publication of any potentially identifiable images or data included in this article.

## Author Contributions

AJ: conceptualization, methodology, investigation, data curation, formal analysis, and writing original draft. ASA, LC, and HL: conceptualization, methodology, and investigation. FA-M: investigation and formal analysis. AA, MA, SA, and NA: investigation. MB: data curation and formal analysis. PT: conceptualization, methodology, investigation, and writing review and editing. All authors contributed to the article and approved the submitted version.

## Conflict of Interest

The authors declare that the research was conducted in the absence of any commercial or financial relationships that could be construed as a potential conflict of interest.

## Publisher's Note

All claims expressed in this article are solely those of the authors and do not necessarily represent those of their affiliated organizations, or those of the publisher, the editors and the reviewers. Any product that may be evaluated in this article, or claim that may be made by its manufacturer, is not guaranteed or endorsed by the publisher.
